# History of Eugenics in Otorhinolaryngology: Ernst Rüdin and the International Eugenics Network

**DOI:** 10.1055/s-0043-1776701

**Published:** 2024-01-24

**Authors:** Beato Suwa

**Affiliations:** 1 Hohendodeleben, Saxony–Anhalt, Germany

**Keywords:** cleft palate, eugenics, history of medicine, national socialism, otosclerosis

## Abstract

**Introduction** The early geneticist and psychiatrist Ernst Rüdin (1874–1952) became one of the key figures in the eugenics movement and in the German health system of the Nazi era. His connections in the international eugenics network have played an important role in the history of eugenics.

**Objective** To discuss the connections between Ernst Rüdin's scientific group in Munich and Otmar von Verschuer's group in Frankfurt during the Nazi era.

**Methods** Otorhinolaryngological materials from Ernst Rüdin's former private library are presented, and they show Rüdin's deep involvement in the international eugenics network. These materials provide insights into early medical genetics in otorhinolaryngology.

**Results** One result of the present study is that eugenics groups from Munich, Frankfurt, and New York certainly influenced one another in the field of otorhinolaryngology. Karlheinz Idelberger and Josef Mengele were two scientists who performed hereditary research on orofacial clefts. Later, Mengele became deeply involved in Nazi medical crimes. His former work on orofacial clefts clearly had, to some extent, an influence on subsequent studies.

**Conclusion** An international eugenics network already existed before 1933. However, it becomes clear that the weaknesses of many early genetic studies did not enable its authors to draw firm scientific conclusions, suggesting that scientists lacked an accurate concept of the genetic causes of most illnesses.

## Introduction

In the German ‘Law for the Prevention of Hereditarily Diseased Offspring’ ( *Gesetz zur Verhütung erbkranken Nachwuchses* ), enacted in January 1934, 2 paragraphs define ‘hereditary deafness’ ( *erbliche Taubheit* ) and ‘severe hereditary malformation’ ( *schwere erbliche körperliche Missbildung* ), including severe forms of combined cleft lip and cleft palate, 1 as reasons for the forced sterilization of these patients. However, the same law also defines a number of disorders, such as ‘congenital feeble-mindedness’ ( *angeborener Schwachsinn* ), ‘shizophrenia’ ( *Schizophrenie* ), and ‘severe alcoholism’ ( *schwerer Alkoholismus* ) as reasons for forced sterilizations. 1

## Ernst Rüdin (1874–1952)

Ernst Rüdin was born on April 19, 1874, in St. Gallen, Switzerland. 2 As a student, he became a follower of Swiss neuroscientist Auguste Forel (1848–1931), a convinced early supporter of forced sterilization. 2 Rüdin began to study medicine and graduated in Zurich on December 2, 1898. 2 He wrote his dissertation and later his postdoctoral lecture qualification thesis on topics related to forensic psychiatry. 2 Rüdin was fond of travelling.

He studied medicine in approximately five different European universities and, after his graduation, he frequently changed his job positions and travelled throughout Switzerland, Berlin, Heidelberg, and Munich. 2 Rüdin was talented in languages and could speak and write in German, English, French, and Italian. 2 Therefore, he had always been an international person. 2 3

In 1909, Rüdin followed his teacher Emil Kraepelin (1856–1926) to Munich University. 2 After the onset of World War I, he was made associate professor at Munich University in 1915. 2 In 1917, he was also appointed director of the Genealogical Demographic Department (GDD), which was part of the German Research Institute for Psychiatry (Deutsche Forschungsanstalt für Psychiatrie, DFA, in German) in Munich, a fairly independent psychiatric research facility. 2 Rüdin was an expert in hereditary studies in psychiatry. 2 3 He became a full professor at Basel University in 1925, but returned to Munich in 1928 and was appointed director of the DFA in 1932. 2 One GDD branch performed research in the field of intelligence tests and diagnostics of intellectual disabilities. 2 After 1933, Rüdin became deeply involved in the Nazi movement. 2 Indeed, he was one of the central figures (if not the main one) responsible for including Nazi concepts such as forced sterilization and later “euthanasia” into German psychiatry. 2 Today, Rüdin's involvement in Nazi crimes is indisputable: he was involved in studies planned to end with the death of the subjects, for example. 2 3 4 Ernst Rüdin passed away in Munich on October 22, 1952. 2 3

## The International Eugenics Network and Ernst Rüdin's Connections to the Eugenics Movement in the United States

Alfred Ploetz (1860–1940), an enthusiastic follower of Auguste Forel, married Ernst Rüdin's sister Pauline Rüdin, one of the first Swiss female physicians, in 1890, and then spent about four years of his life in the United States in the 1890s. 2 One of the founders of the German race hygiene ( *Rassenhygiene* ) movement, Alfred Ploetz had an enormous influence on Ernst Rüdin. 2 3 In 1904, Ernst Rüdin became editor of the *Archiv für Rassen-und Gesellschaftsbiologie* ( *Archive for Racial and Social Biology* ) journal, founded by Ploetz. 2 His work as an editor led to many international voyages and meetings with different international (mostly European) scientists. 2 Furthermore, Ernst Rüdin was introduced by Ploetz to many important international personalities who were part of the hygiene movement and the early eugenics movement. 2 Together, Ploetz and Ernst Rüdin founded the Society for Race Hygiene (Gesellschaft für Rassenhygiene) in 1905, 2 which already partly operated internationally. 2 In the United States, Indiana enacted the first sterilization law in 1907 and, by the mid 1930s, over half of the states in the country had passed laws authorizing forced sterilization. 5 In 1912, a Permanent International Eugenics Committee was founded in Great Britain. 6 In 1921, after World War I, this committee continued its work, and was renamed the International Federation of Eugenics Organizations (IFEO) in 1925. 6 Charles Davenport, of the Eugenics Record Office at Cold Spring Harbor Laboratory (Long Island), was a key figure, becoming IFEO president between 1928 and 1932. 6 Ernst Rüdin became a member of the IFEO on September 27, 1929, after a recommendation by Ploetz. 2 In December 1929, the IFEO organized a meeting in Munich, and Ernst Rüdin held a lecture on psychiatry and race hygiene. 2 He also contributed two lectures to the IFEO congress in Farnham (Dorset, United Kingdom) in 1930. 2 In 1932, Ernst Rüdin was elected IFEO president at its congress in New York (he did not attend it personally). 2 In 1933, the Nazis came to power, leading to significant problems for the IFEO. 6 In the following years, German influence dominated the IFEO, leading many independent suborganizations and individuals to withdraw. 6 These problems became particularly apparent at the 1934 Congress in Zurich, Switzerland (Rüdin's home country), and the 1936 congress in The Netherlands. 6 As a result of these problems, the IFEO was dissolved in the late 1930s. 6 Another result of Nazi politics and World War II was that many countries, including the United States, changed their views on eugenics and began to reject forced sterilizations. 5 6

## Genetics of Otosclerosis

Today, we know that both genetic and environmental factors contribute to the development of otosclerosis, a complex genetic disease 7 8 whose inheritance pattern is considered autosomal dominant with reduced penetrance, with 40% to 50% of cases appearing to be sporadic. 8 9 Various environmental factors are associated with otosclerosis, including persistant measles virus infection and estrogen exposure. Additionally, sodium fluoride exposure (through fluoridated water) is associated with protection against otosclerosis. However, these associations lack sufficient evidence for causality. 8

## Genetics of the Cleft Palate

Today, cleft palates should be divided into non-syndromic and syndromic clefts. 10 Most syndromic clefts, which present with other physical or cognitive abnormalities, have a known genetic cause, while nonsyndromic clefts are believed to be caused by gene–environment interactions. 10 Many genes have been associated with nonsyndromic cleft palate in recent years. 10 The environmental factors associated with non-syndromic cleft palate include maternal smoking and alcohol abuse during pregnancy. 11 12 Nutrition during pregnancy, especially the ingestion of folate, might also play an important role. 13

## Objective

The present study discusses the connections between Ernst Rüdin's scientific group in Munich and Otmar von Verschuer's group in Frankfurt during the Nazi era, and it shows that eugenic groups from Munich, Frankfurt, and New York certainly influenced one another in the field of otorhinolaryngology.

## Materials and Methods

The document analysis method in qualitative research was used in the present study. Historical materials written in German were carefully translated in parts into English. The contents of the materials were analyzed by comparing them with different references and previous studies. Step by step, the historical context was discussed, resulting in an overview of the presented topic. Furthermore, recent studies on similar topics were analysed and discussed in connection with the materials.

**Fig. 1:**
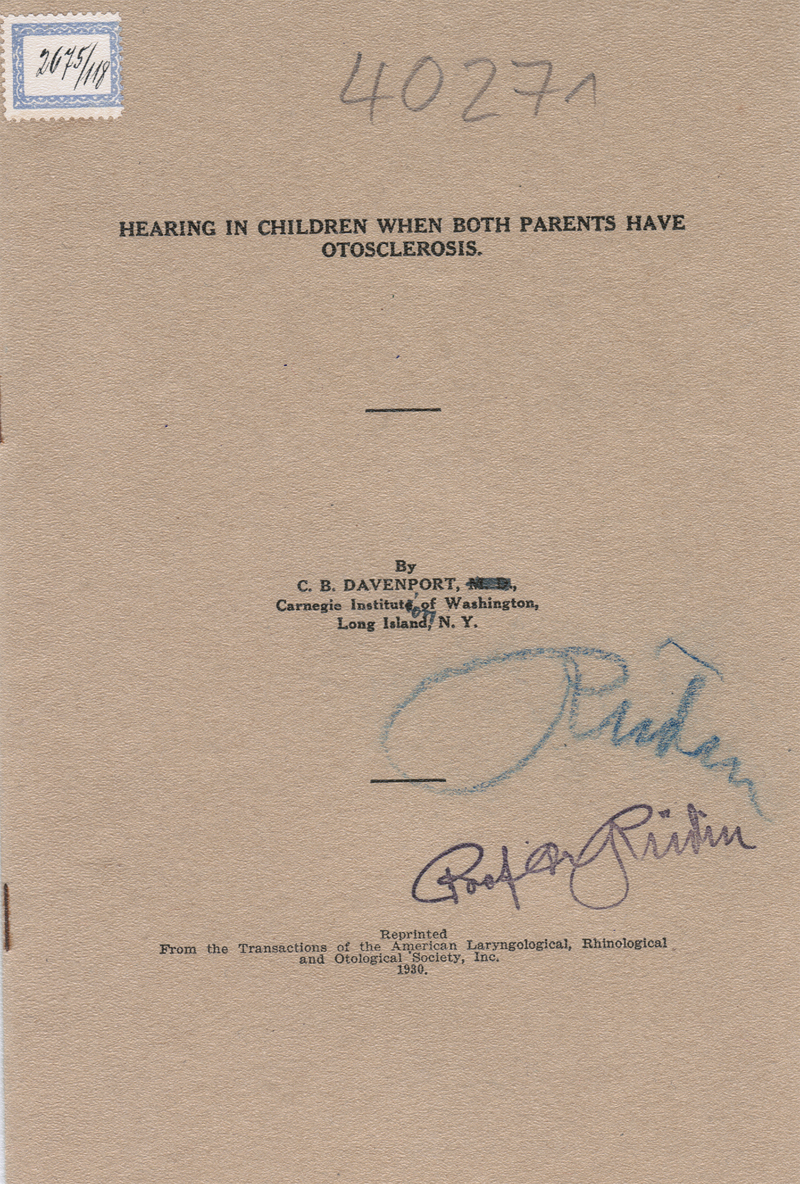
‘Hearing in Children when both Parents have Otosclerosis’, by C. B. Davenport. 14

**Fig. 2:**
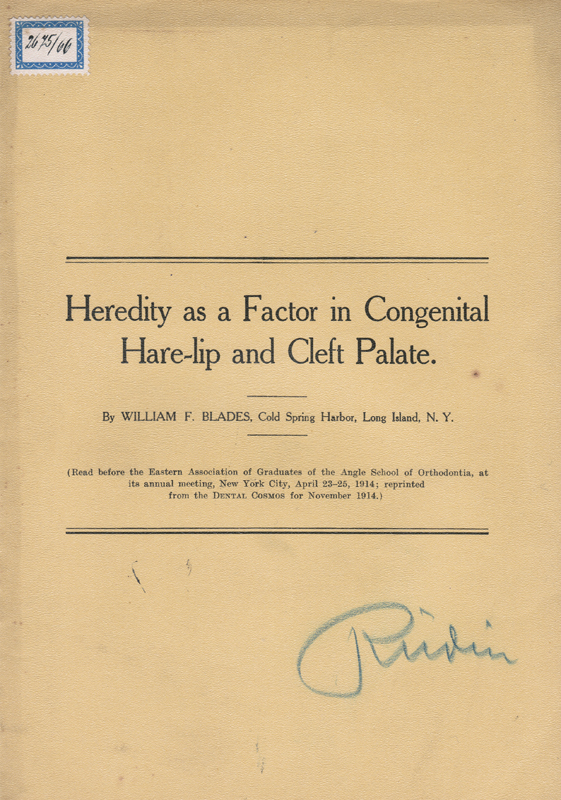
‘Heredity as a Factor in Congenital Hare-lip and Cleft Palate’, by William F. Blades. 15

**Fig. 3:**
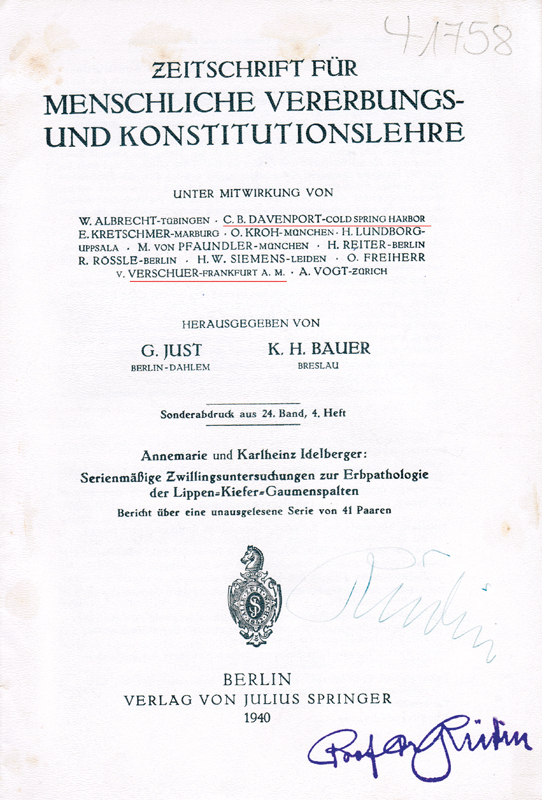
‘Series of twin studies on the hereditary pathology of cleft lip and palate’, by A. Idelberger and K. Idelberger. 16

The following materials from Ernst Rüdin's library are part of the present study:

Material 1: ‘Hearing in Children when both Parents have Otosclerosis’, by C. B. Davenport 14 ( Fig. 1 ).
Material 2: ‘Heredity as a Factor in Congenital Hare-lip and Cleft Palate’, by William F. Blades 15 ( Fig. 2 ).
Material 3: ‘Series of twin studies on the hereditary pathology of cleft lip and palate’, by A. Idelberger and K. Idelberger 16 ( Fig. 3 ) ( Supplementary Material File 1 ). 17
Material 4: ‘Examinations of the teeth of Allgäu full cretins with particular attention to the question of caries’, by K. Schenkel ( Supplementary Material File 2 ). 18
Material 5: ‘On carcinomatous meningitis’, by E. Schlittler ( Supplementary Material File 3 ). 19

Parts of Ernst Rüdin's library, including the materials herein discussed, were donated to the archive of the Ludwig Maximilian University of Munich. 20

All materials were carefully evaluated, and their authenticity could be verified. The fact that many related materials were also part of Ernst Rüdin's library is an important indication of its authenticity.

The complete line of provenance could be followed for the materials from approximately 1926 until today. The widower of Ernst Rüdin's only daughter passed away in 2020 at a very advanced age (the materials were part of his possessions). Therefore, all original materials presented can be considered ‘novel materials’ in the sense that they were not part of a detailed historical analysis before.

## Results

Material 1: 14 Seven different family trees were discussed in this study, three of which were very small and only showed two generations. Davenport supports the theory of a recessive background for otosclerosis, but also suggests that there could be another type of otosclerosis with dominant background (‘The view, therefore, that there are two types of otosclerosis, one of which depends upon a dominant gene and the other upon a recessive one, is a priori not improbable.’). 14

Material 2: 15 Only two different family trees are shown in this study. 15 Therefore, the author was cautious in his conclusions, and merely suggested heredity as one factor in congenital ‘hare-lip’ and ‘cleft palate’. 15

Material 3: 16 This study was performed in the late 1930s, and it included data from 41 pairs of identical and fraternal twins. 16 Otmar von Verschuer (1896–1969) and his former assistant at Frankfurt University, Josef Mengele (1911–1979), are mentioned several times in this article. 16 21 In Nazi Germany, Verschuer had specialized in twin studies in particular. 22 Mengele's medical dissertation was titled ‘Heredity of combined cleft lip, cleft maxilla and cleft palate’, 23 and it was published in the *Journal for Human Heredity and Constitution* , the same journal that published material 3, 16 which had Charles Davenport and Verschuer among its contributors ( Fig. 3 ). In the 1980s, many articles 24 25 were published on Mengele's deep involvement in Nazi medical crimes during World War II. The article by A. and K. Idelberger 16 cites Mengele's dissertation as a reference on page 479 and mentions it on pages 420 and 421 ( Fig. 3 ). Preliminary results of the study by A. and K. Idelberger were presented at the 34th congress of the German Orthopaedic Society in Berlin between September 16 and 18, 1940 ( Supplementary Material File 1 ). 17 Karlheinz Idelberger (1909–2003) later became an influential orthopedist in Germany. 26 A. and K. Idelberger 16 frequently referred to a study written in English by Sanders 27 in 1934 and to Mengele's dissertation. 23

Material 4: 18 The study was performed at the Munich University's Dental Clinic in cooperation with Ernst Rüdin's GDD. The author mentions that it was part of a broader series of studies on goiter, ‘cretinism’ ( *Kretinismus* ), palatine abnormalities, ‘deaf-mutism’ ( *Taubstummheit* ) and ‘feeble-mindedness’ ( *Schwachsinn* ) in the Bavarian Allgäu Mountains organized by Ernst Rüdin's GDD and assigned by the Bavarian State Government. 18 (The German word *Vollkretinen* refers to severe abnormalities in the physical and intellectual development of children, mainly caused by iodine deficiency, which are currently called hypothyroidism)

Material 5: 19 Emil Schlittler (ca.1879–1949) had been Ernst Oppikofer's deputy at Basel University's Clinic of Otorhinolaryngology. 28 29 Schlittler's study 19 was published during Ernst Rüdin's time as a full professor of Psychiatry at Basel University. 2 3

## Discussion

Material 1: 14 Today we know that otosclerosis has a complex genetic background and an inheritance pattern considered autosomal dominant, with reduced penetrance. 8 It becomes clear that Davenport did not have an accurate concept of the genetic background of otosclerosis around the year 1930, partly because there was not enough statistical data on the condition. 14

Material 2: 15 The work clearly shows that scientific research about the heredity of clefts was not only conducted in Germany or elsewhere in Europe, but also in the United States. Furthermore, one copy of the study 15 certainly was part of Ernst Rüdin's library. Therefore, we can assume that Rüdin and his group in Munich could have read the study, and we can get an idea how the different groups in the international eugenics network might have influenced each other.

Material 3: 16 One of the central points of Mengele's medical dissertation 23 can be seen in the so-called ‘microforms’ ( *Mikroformen* ) of combined cleft lip, cleft maxilla, and cleft palate ( *Lippen-Kiefer-Gaumenspalte* ). Mengele postulated that malformations of one of the two second upper dens incisivi or the two upper dens canini, morphological palate malformations, submucus cleft palate, slight morphological malformations of the upper lip, and bifid (very small malformations in the sense of) partly divided or cleft uvula (minor manifestations of bifid uvula) should be defined as ‘microforms’, and that there was one single ‘dysregular dominant heredity’ ( *unregelmäßig dominanter Erbgang* , roughly comparable to today's *autosomal dominant pattern* with reduced penetrance) for all forms of cleft lip, cleft maxilla, cleft palate, and all ‘microforms’ together. 23 Today we know that the heredity of these conditions is far more complicated. 10

A. and K. Idelberger 16 criticized Mengele's dissertation by writing that the connections involving malformations of the uvula or tooth position and cleft lip, cleft maxilla, and cleft palate would require further investigations before they could be accepted as a proof that isolated clefts are hereditary. Furthermore, they indirectly criticized Mengele by writing that German surgeon Carl Stobwasser had already postulated a connection between the heredity of cleft lips ( *Hasenscharten* ) and dental malformations (Stobwasser also mentioned one patient with combined double-sided cleft lip and double-sided coloboma iridis) in 1884. They even wrote that Stobwasser – and explicitly not Mengele or Schröder (in 1931, Schröder published a work about ‘The heredity of cleft lip and cleft palate in 21 pedigrees’ in Ernst Rüdin's *Archive for Racial and Social Biology* journal)– had been the first to describe the ‘microforms’, although Stobwasser did not use the word ‘microforms’ in 1884. 16 23 30 31 Stobwasser 30 reported about 6 cases of dental malformations in patients simultaneously diagnosed with cleft lip.

The extremely critical attitude of A. and K. Idelberger 16 towards Mengele's medical dissertation 23 might be considered a sign of rivalry between Verschuer's group in Frankfurt and Rüdin's group in Munich. 2 The dissertation only included 14 patients with combined cleft lip, cleft maxilla, and cleft palate, 3 patients with less severe forms, and the families of those 17 patients (Mengele wrote that medical data from 746 different individuals were considered, and that he personally examined 583 individuals). 21 23 While Mengele's conclusions are questionable, the scientific impact of his medical dissertation is, to some extent, indisputable and, surprisingly, can also be seen after World War II. 16 21 23 32 33 Benzenhöfer and Weiske 21 (2010) reported that a Japanese study from 1970 and a British study from 1972 even cited the dissertation (both studies cited it by writing Mengele's name incorrectly). 32 33 Furthermore, they analyzed the dissertation in detail and suggested that Mengele might have included patients with ‘microforms’ in his dissertation to achieve a higher percentage of hereditary cases for his study. 21 Nevertheless, they did not mention the study by the Idelbergers, 16 neither did they focus on recent definitions of the term ‘microforms’. 21

From 1945 to 1992 approximately, several studies still used the term *microforms* , in a way similar to its use by Mengele, to refer to dental malformations, cleft palate, and cleft uvula. 34 35 In the last 30 years, many studies 36 37 38 have actually used *microforms* exclusively to describe very small clefts of the upper lip. Small, but not very small, malformations are often distinguished with the term *mini - microform cleft* . 39 40 The term *mini - microform cleft* is partly used to distinguish extremely small malformations or clefts. 41 One Japanese case report presented a patient with ‘mini-microform cleft’ who did not show sonographic evidence of orbicularis oris muscle (OOM) rupture; his diagnosis was based on threedimensional facial measurements. 42 Nevertheless, such *microforms* may be connected to asymmetry and functional problems of the nostrils and the nose. 34 43 The definitions of the term microform still are not exactly and consistently given in different studies of the last 30 years. Most recent studies, but not all studies, only use the term in connection with cleft lip. With a few exceptions, 44 45 over the past 30 years, the term has not been used frequently in connection with other craniofacial malformations (other than cleft lip). One such exception is holoprosencephaly (HPE); 46 47 a very interesting aspect is discussed in one study: 48 several specific mutations causing clear phenotypic signs of “microform HPE” could be linked with above average intellectual function. 48

It is important to emphasize that most recent studies did not use the term in the same sense intended by Mengele. Nevertheless, its frequent use in the last 30 years shows that Mengele's dissertation (and also Schröder's work from 1931) surely had a certain scientific impact around 1940, and at least indirectly, still has a certain influence on today's scientific publications in the field of craniofacial clefts. The fact that the common definition of the term has obviously developed and changed in the past 80 years is also a clear indication of Mengele's (and Schröder's) scientific influence. Therefore, the different (partly historical) definitions *microform* or *microforms* should be distinguished clearly, the intended meaning should be defined accuratetly, and the terms should not be used without care.

In 1960 and 1961, Mengele lost both doctoral degrees (his medical degree and his philosophical/anthropological degree) because of his participation and deep involvement in Nazi medical crimes. 49 After World War II, in July 1949, Mengele fled to South America: first, to Argentina; then, he fled to Paraguay in 1959 and, in 1960, to Brazil, to the towns of Nova Europa, Caieiras, and Diadema, located in the state of São Paulo. He lived almost 20 years in Brazil until he died, on February 7, 1979. 24 25 49 50

Today, congenital conditions such as cleft palate or congenital dysplasia of the hip (CDH) are treatable and curable, but they were partly considered ‘severe hereditary malformations’ ( *schwere erbliche, körperliche Missbildung* ) in Nazi Germany. 1 50 An article written by Rüdin's group in Munich titled ‘CDH as a severe physical malformation’ (‘Hüftverrenkung als schwere körperliche Missbildungen’) was part of Ernst Rüdin's library. 20 In one copy of that article, there are certain interesting marks and underlined excerpts, which were probably made by Ernst Rüdin himself, and that copy was part of the materials donated to the archive of the Ludwig Maximilian University of Munich in 2021. 20

In the official comment of the ‘Law for the Prevention of Hereditarily Diseased Offspring’, Ernst Rüdin and his coauthors emphasized the hereditary character of diseases. Some of the considered reasons to ‘implement forced sterilization’ are treatable and curable today (such as severe cleft palate). 1 The methodology of the study by A. and K. Idelberger 16 should be considered advanced and innovative, given that it was published in 1940.

Material 4: 18 The study mainly comprised data on caries in dental medicine, but otorhinolaryngological data were also included in it. This study's 18 methodology is notable for its comparably ‘high’ number of cases, given that it was published in 1932.

Material 5: 19 The fact this article is part of Ernst Rüdin's library clearly indicates that he had established scientific relationships with otolaryngologists during his time at Basel University (1925–1928). 2 3

## Conclusion

Within the international eugenics network, the groups surrounding Ernst Rüdin (Munich), Otmar von Verschuer (Frankfurt), and Charles Davenport (New York) significantly influenced one another, even in the scientific field of otorhinolaryngology.

Karlheinz Idelberger (Munich) and Josef Mengele (Frankfurt) were two notable scientists who performed hereditary research on ‘cleft lip’ and ‘cleft palate’ before Mengele became deeply involved in Nazi medical crimes.

Furthermore, we can conclude that Stobwasser's ‘minor conditions’ (dental malformations and coloboma iridis) were not completely identical to Mengele's ‘microforms’, as indicated by A. and K. Idelberger. 16 23 30

It becomes clear that the methodological weaknesses of many early genetic studies did not enable their authors to draw firm scientific conclusions, suggesting that scientists did not have an accurate concept of the genetic causes of most illnesses.

Until approximately 1945, the international eugenics movement was a central part of the medical scientific community; therefore, it certainly had a significant influence on most subfields of medicine, including otorhinolaryngology. Due to the fact that eugenics was not only based on natural science, but also based on political ideology to some extent, the eugenic approaches in medicine were often characterized by scientific inaccuracies and mistakes (at least from the perspective of the current times). One of the main theses of the present work is that after 1945, the influence of eugenics may have persisted to some extent as very subtle factors (such as in the diagnostic term *microform* ) in otorhinolaryngology, and even today it may still hold a certain influence, both on clinical medicine and on medical science. We should become sensitive to this issue and try to define our diagnoses as accurately as possible.
