# Supplementary material for: History of Eugenics in Otorhinolaryngology: Ernst Rüdin and the International Eugenics Network
Source: Int Arch Otorhinolaryngol. 2024 Jan 24;28(2):e319–25. doi: 10.1055/s-0043-1776701 (PMC11008944; doi:10.1055/s-0043-1776701)
Supplement: Supplementary file 1 — Supplementary Material [file 10-1055-s-0043-1776701-s2023021486or.pdf]

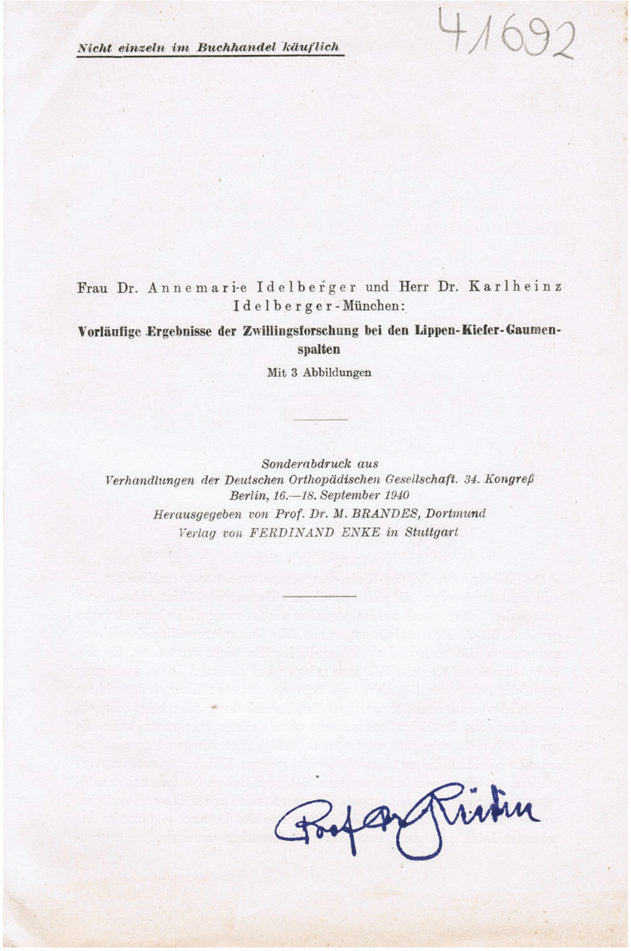

Nicht einzeln im Buchhandel käuflich

41692

Frau Dr. Annemarie Idelberger und Herr Dr. Karlheinz Idelberger-München:

Vorläufige Ergebnisse der Zwillingsforschung bei den Lippen-Kiefer-Gaumen-spalten

Mit 3 Abbildungen

Sonderabdruck aus
Verhandlungen der Deutschen Orthopädischen Gesellschaft. 34. Kongreß
Berlin, 16.—18. September 1940
Herausgegeben von Prof. Dr. M. BRANDES, Dortmund
Verlag von FERDINAND ENKE in Stuttgart

Prof Dr Rüdin

Supplementary Material File 1 'Preliminary results of twin research on cleft lip and palate', by A. Idelberger and K. Idelberger.[17]

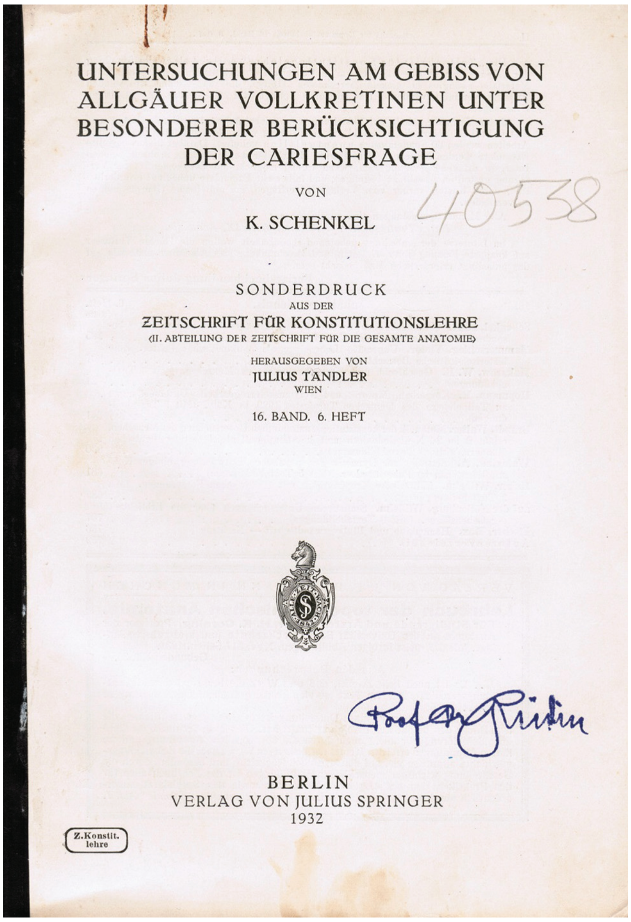

UNTERSUCHUNGEN AM GEBISS VON ALLGÄUER VOLLKRETINEN UNTER BESONDERER BERÜCKSICHTIGUNG DER CARIESFRAGE

VON

K. SCHENKEL

40538

SONDERDRUCK
AUS DER
ZEITSCHRIFT FÜR KONSTITUTIONSLEHRE
(II. ABTEILUNG DER ZEITSCHRIFT FÜR DIE GESAMTE ANATOMIE)
HERAUSGEGEBEN VON
JULIUS TANDLER
WIEN

16. BAND. 6. HEFT

Prof Dr Rüdin

BERLIN
VERLAG VON JULIUS SPRINGER
1932

Z.Konstit. lehre

Supplementary Material File 2 'Examinations of the teeth of Allgäu full cretins with particular attention to the question of caries', by K. Schenkel.[18]

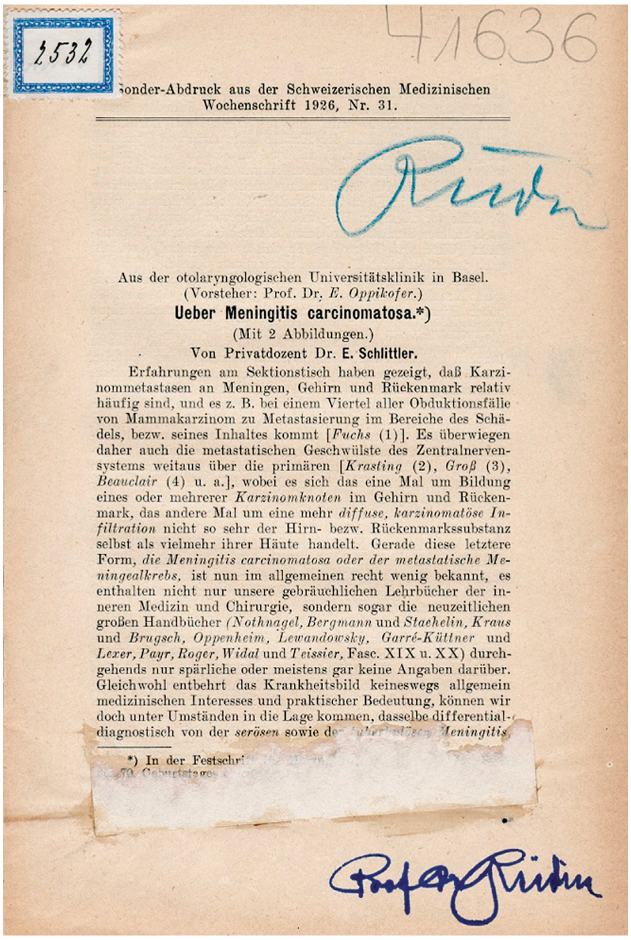

2532

41636

Sonder-Abdruck aus der Schweizerischen Medizinischen Wochenschrift 1926, Nr. 31.

Rüdin

Aus der otolaryngologischen Universitätsklinik in Basel.
(Vorsteher: Prof. Dr. *E. Oppikofer.*)

Ueber Meningitis carcinomatosa.*)

(Mit 2 Abbildungen.)

Von Privatdozent Dr. **E. Schlittler.**

Erfahrungen am Sektionstisch haben gezeigt, daß Karzinommetastasen an Meningen, Gehirn und Rückenmark relativ häufig sind, und es z. B. bei einem Viertel aller Obduktionsfälle von Mammakarzinom zu Metastasierung im Bereiche des Schädels, bezw. seines Inhaltes kommt [*Fuchs* (1)]. Es überwiegen daher auch die metastatischen Geschwülste des Zentralnervensystems weitaus über die primären [*Krasting* (2), *Groß* (3), *Beauclair* (4) u. a.], wobei es sich das eine Mal um Bildung eines oder mehrerer *Karzinomknoten* im Gehirn und Rückenmark, das andere Mal um eine mehr *diffuse, karzinomatöse Infiltration* nicht so sehr der Hirn- bezw. Rückenmarkssubstanz selbst als vielmehr ihrer Häute handelt. Gerade diese letztere Form, *die Meningitis carcinomatosa oder der metastatische Meningealkrebs,* ist nun im allgemeinen recht wenig bekannt, es enthalten nicht nur unsere gebräuchlichen Lehrbücher der inneren Medizin und Chirurgie, sondern sogar die neuzeitlichen großen Handbücher (*Nothnagel, Bergmann* und *Staehelin, Kraus* und *Brugsch, Oppenheim, Lewandowsky, Garré-Küttner* und *Lexer, Payr, Roger, Widal* und *Teissier,* Fasc. XIX u. XX) durchgehends nur spärliche oder meistens gar keine Angaben darüber. Gleichwohl entbehrt das Krankheitsbild keineswegs allgemein medizinischen Interesses und praktischer Bedeutung, können wir doch unter Umständen in die Lage kommen, dasselbe differentialdiagnostisch von der *serösen* sowie de[illegible] *Meningitis,*

*) In der Festschri[illegible]
[illegible] 70. Geburtstages [illegible]

Prof. Dr. Rüdin

Supplementary Material File 3 'On carcinomatous meningitis', by E. Schlittler.[19]
